# 合成酚类抗氧化剂的环境赋存特征、生态风险及分析方法研究进展

**DOI:** 10.3724/SP.J.1123.2026.02013

**Published:** 2026-09-08

**Authors:** Sisi LIU, Zijun LI, Hongbo WEI, Piaoli XU, Lina LI, Changer CHEN, Guangguo YING

**Affiliations:** 华南师范大学环境研究院/环境学院，广东省化学污染与环境安全重点实验室，环境理论化学教育部重点实验室，广东 广州 510006; School of Environment/Environmental Research Institute，South China Normal University，Guangdong Provincial Key Laboratory of Chemical Pollution and Environmental Safety，MOE Key Laboratory of Theoretical Chemistry of Environment，Guangzhou 510006，China

**Keywords:** 合成酚类抗氧化剂, 环境赋存, 生态风险, 环境分析, 非靶向筛查, 转化产物, synthetic phenolic antioxidants （SPAs）, environmental occurrence, ecological risk, environmental analysis, non-target screening, transformation products

## Abstract

合成酚类抗氧化剂（SPAs）是一类能够有效延缓高分子材料老化的化学助剂，已被广泛应用在塑料、橡胶及食品包装等领域。随着新型SPAs在各种环境介质中被不断检出，其环境健康与生态风险日益凸显。本文重点综述了近5年SPAs在水体、沉积物、土壤、室内灰尘及生物体等环境介质中的赋存特征，对照过往数据，SPAs的污染特征呈现从传统SPAs向新型高相对分子质量SPAs转变的趋势。本文还总结了SPAs样品前处理技术（如超声提取、固相萃取）的优化策略，以及基于大气压化学电离源（APCI）的液相色谱-串联质谱和高分辨质谱非靶向筛查技术在SPAs痕量分析与未知代谢物识别中的应用进展。最后，本文总结了SPAs及其转化产物的环境健康与生态风险，指出了当前研究在环境分析筛查方法、转化产物与新型SPAs的风险以及环境行为与毒理学机制等方面的不足，并展望了未来的研究方向，旨在为SPAs的全面风险评估提供科学依据。

## Abstract (en)

Synthetic phenolic antioxidants （SPAs） are a major class of chemical additives extensively employed in plastics， rubber， and food packaging to prevent polymer degradation and extend product lifespans. In recent years， the environmental and human health risks of SPAs have received great concern due to their widespread occurrence in various environmental media. Existing reviews primarily focus on environmental detection， human exposure， toxicity， and risk assessment of SPAs with limited discussion on recent advances in analytical methodologies. This review presents a systematic synthesis of research advances over the past five years， especially the state of the art analytical methodologies. This review systematically analyzes their occurrence in water， sludge， sediments， soils， dust， and biota tissues， revealing a clear pollution transition from conventional SPAs to emerging high-molecular-weight substitutes. Toxicological evidence demonstrates that SPAs exert adverse effects on rats， mice， and aquatic organisms， including genotoxicity， immunotoxicity， endocrine disruption， reproductive toxicity， etc. Notably， certain transformation products （TPs） of butylated hydroxytoluene （BHT） exhibit enhanced toxicity relative to their parent compounds. Methodological advances in sample preparation and analysis techniques of SPAs in different environmental matrices are reviewed for the first time. Recent applications of atmospheric pressure chemical ionization coupled with liquid chromatography-tandem mass spectrometry （APCI-LC-MS/MS）， as well as high-resolution mass spectrometry （HRMS） provide powerful approaches for target/non-target analysis of unknown SPA derivatives and TPs. Several critical research gaps have finally been identified. Environmental analytical methods of SPAs and their TPs face significant challenges due to their diverse physicochemical properties and chemical reactivity， particularly their susceptibility to oxidation. Another key issue is that current pretreatment approaches for SPAs in tissue samples overlook bound-SPAs， thereby underestimating their real body burdens. Moreover， existing studies mainly focus on traditional SPAs， while lacking systematic assessment of environmental fate and long-term ecological risks posed by emerging SPAs and potentially more toxic TPs. Additionally， ecotoxicological data of SPAs for terrestrial organisms are scarce compared to aquatic organisms and mammals， limiting comprehensive ecological risk assessment. Future research should prioritize： （1） developing oxidant-resistant pretreatment methodologies coupled with HRMS to overcome technical challenges in accurate quantification of trace SPAs and their bound species； （2） strengthening non-target screening and identification of emerging SPAs and TPs； （3） investigating long-term effects of SPAs in soil-plant-microorganism systems. This review advances our understanding of SPAs by bridging the knowledge gaps and providing updated insights into current research. It will help establish the scientific basis for comprehensive ecological risk assessment of SPAs.

## 1 合成酚类抗氧化剂的使用

合成抗氧化剂是一类可显著延缓高分子聚合材料氧化降解过程的化学添加剂，全球市场规模庞大，其中合成酚类抗氧化剂（SPAs）使用量最大、应用范围最广^［1］^。据估算，2023年中国抗氧化剂的产量为144 700 t，其中SPAs的使用量占比接近50%^［1］^。与其他类型合成抗氧化剂相比，SPAs含有一个或多个受阻苯酚类结构，具有较低毒性、不易变色、高相容性和耐热性。因此，SPAs可以作为主抗氧化剂被广泛添加到食品接触材料、橡胶、塑料制品和燃料等高分子聚合材料中。目前商品化SPAs主要包括受阻酚类、双酚类和硫代酯类。各类SPAs的抗氧化机理和环境稳定性因其结构特征存在显著差异。其中，2，6-二叔丁基-4-甲基苯酚（BHT）的使用时间最早、范围最广^［2］^。BHT在中国的年平均产量约9 000 t^［3］^。此外，大量具有更高相对分子质量的新型SPAs正被不断合成、使用。它们多是BHT的高相对分子质量衍生物，具有相同的母体和不同的空间位阻结构。代表性的新型SPAs有2，4，6-三叔丁基苯酚（AO246）、四［3-（3，5-二叔丁基-4-羟基苯基）丙酸］季戊四醇酯（AO1010）、1，3，5-三（3，5-二叔丁基-4-羟基苄基）异氰尿酸（AO3114）和3-（3，5-二叔丁基-4-羟基苯基）丙酸十八烷基酯（AO1076）等。

大多数SPAs以游离态形式存在于聚合物内，无化学键连接，易从塑料中析出并进入环境。从其理化性质来看（表1），SPAs为疏水性难电离化合物（log *K*_OW_：2.84~19.60，p*K*_a_：10~12），挥发性低（log *K*_OA_：7.95~39.35），其理论生物富集因子（BCF）最高可达2 776 L/kg（AO246），具有较强的生物富集潜力。根据其结构和预测的降解半衰期判断，SPAs在空气和水中不稳定，易被氧化。目前，SPAs在各类环境介质中被广泛检出。有必要开展SPAs

**表1:** 典型合成酚类抗氧化剂的物理化学性质^*^

Synthetic phenolic antioxidant	Abbreviation	Structure	Water solubility/（mg/L）	BCF*/*（L/kg wet weight）	log *K*_OW_	log *K*_OA_	Half-life /d
4-*tert*-Octylphenol	4-tOP	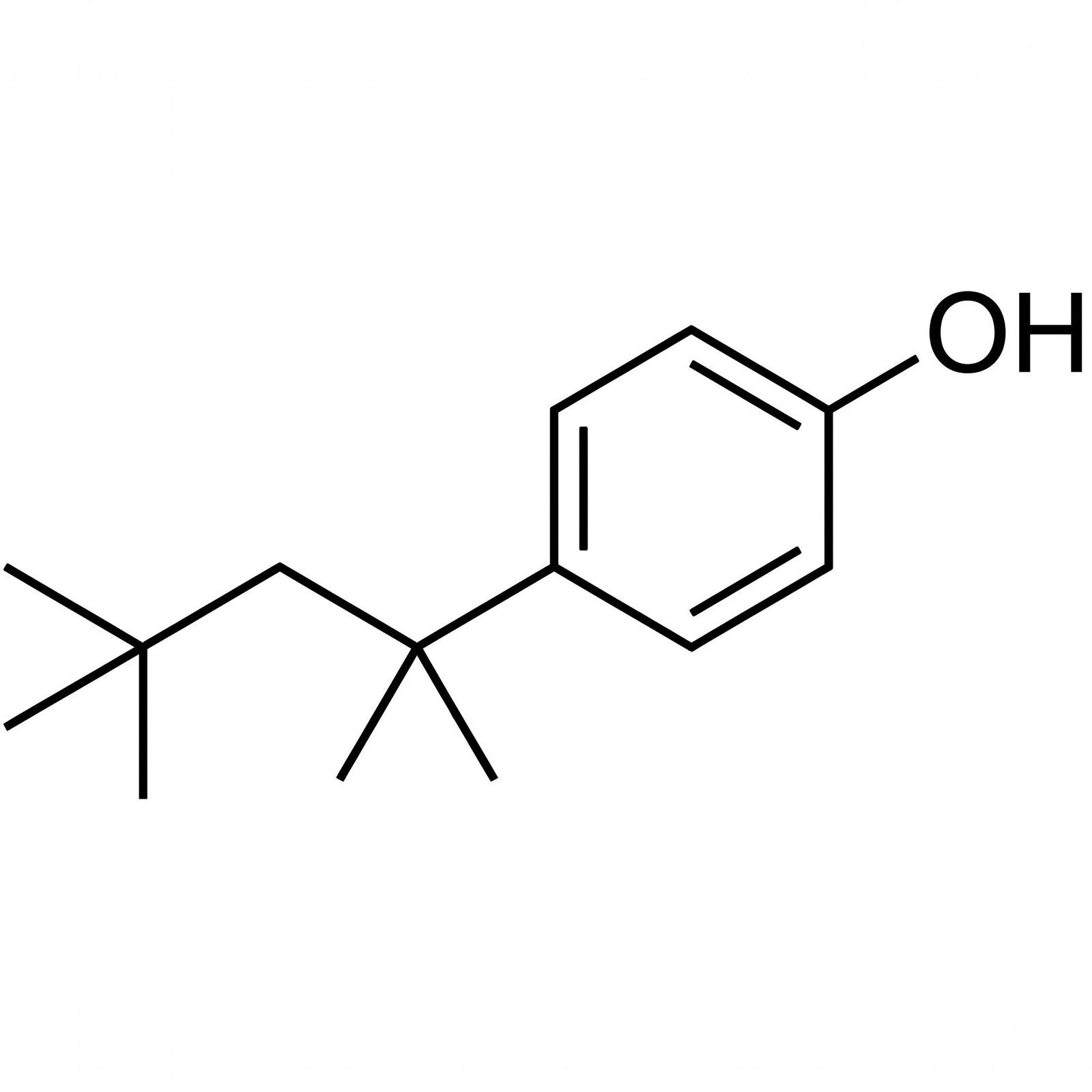	4.8	1406	5.28	9.02	0.25
2，4，6-Tri-*tert*-butylphenol	AO246	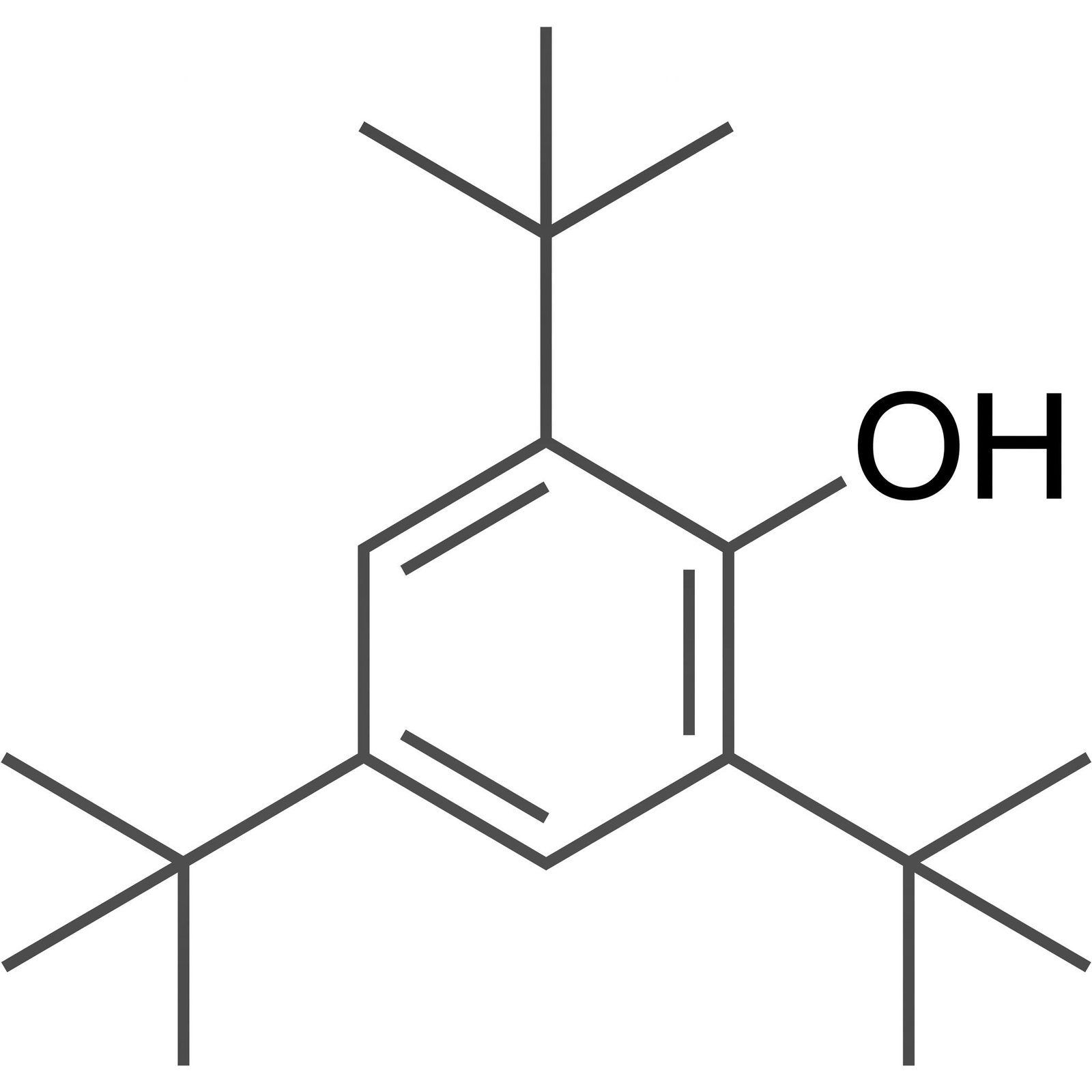	35.0	2776	6.39	9.46	0.67
4，4-Thiobis（6-*tert*-butyl-*m*-cresol）	AO300	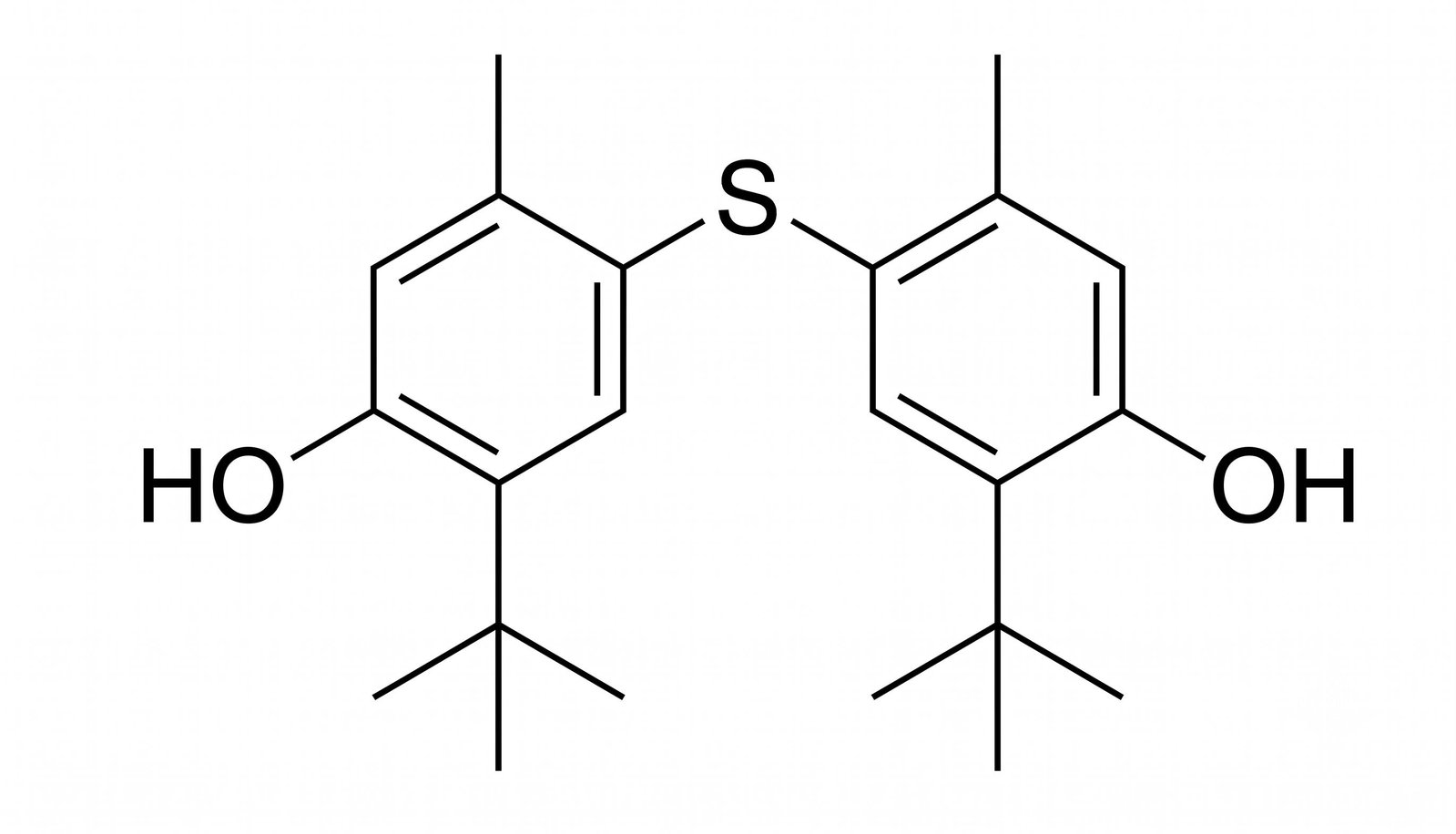	0.0019	1961	8.24	18.19	0.082
2-*tert*-Butyl-4-methoxyphenol	BHA	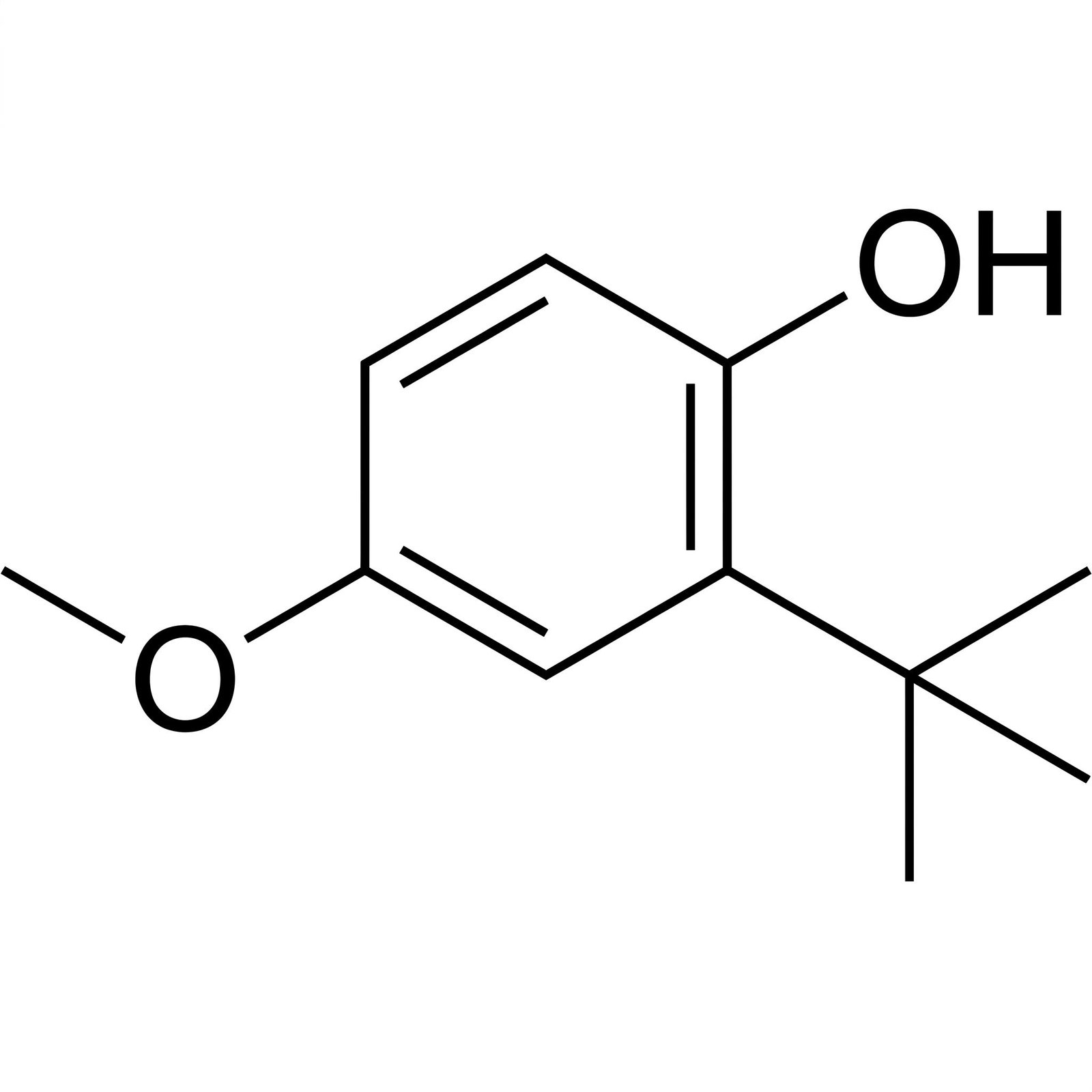	743	57	3.50	8.96	0.30
2，6-di-*tert*-butyl-4-methylphenol	BHT	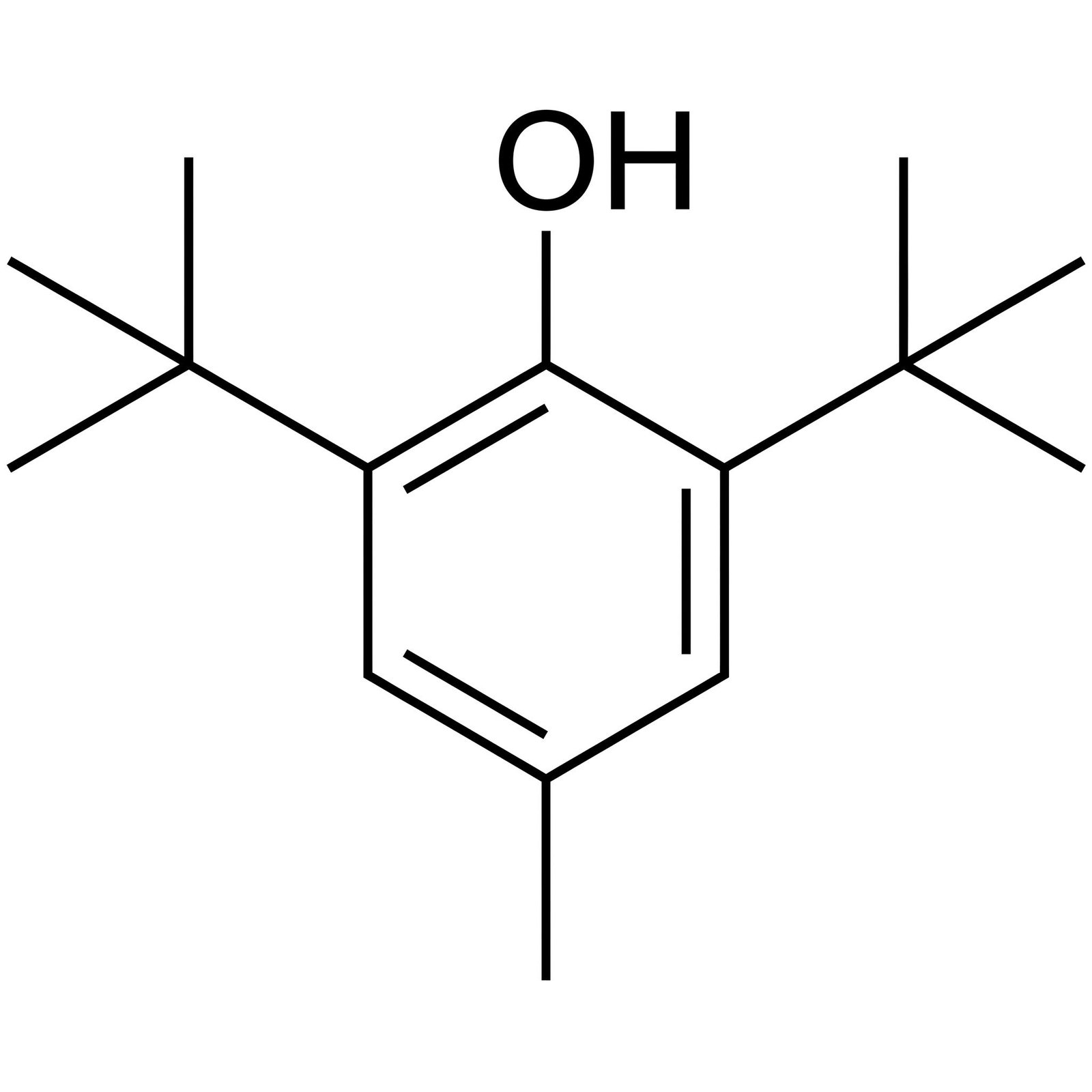	19.4	2.8	5.10	8.87	0.59
2，6-Di-*tert*-butyl-*p*-benzoquinone	BHT-Q	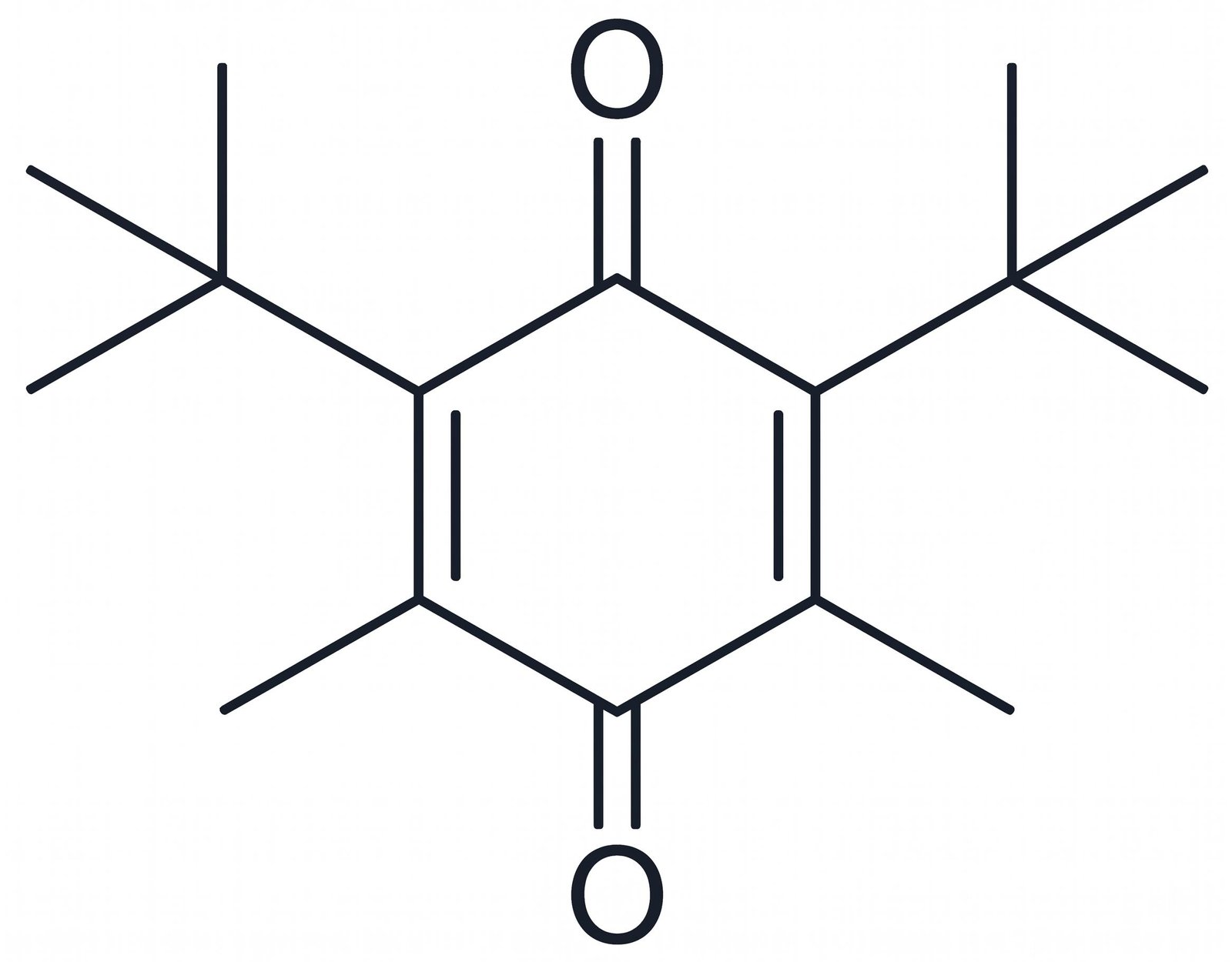	3.5	18.2	4.42	7.95	172
3，5-Di-*tert*-butyl-4-hydroxybenzaldehyde	BHT-CHO	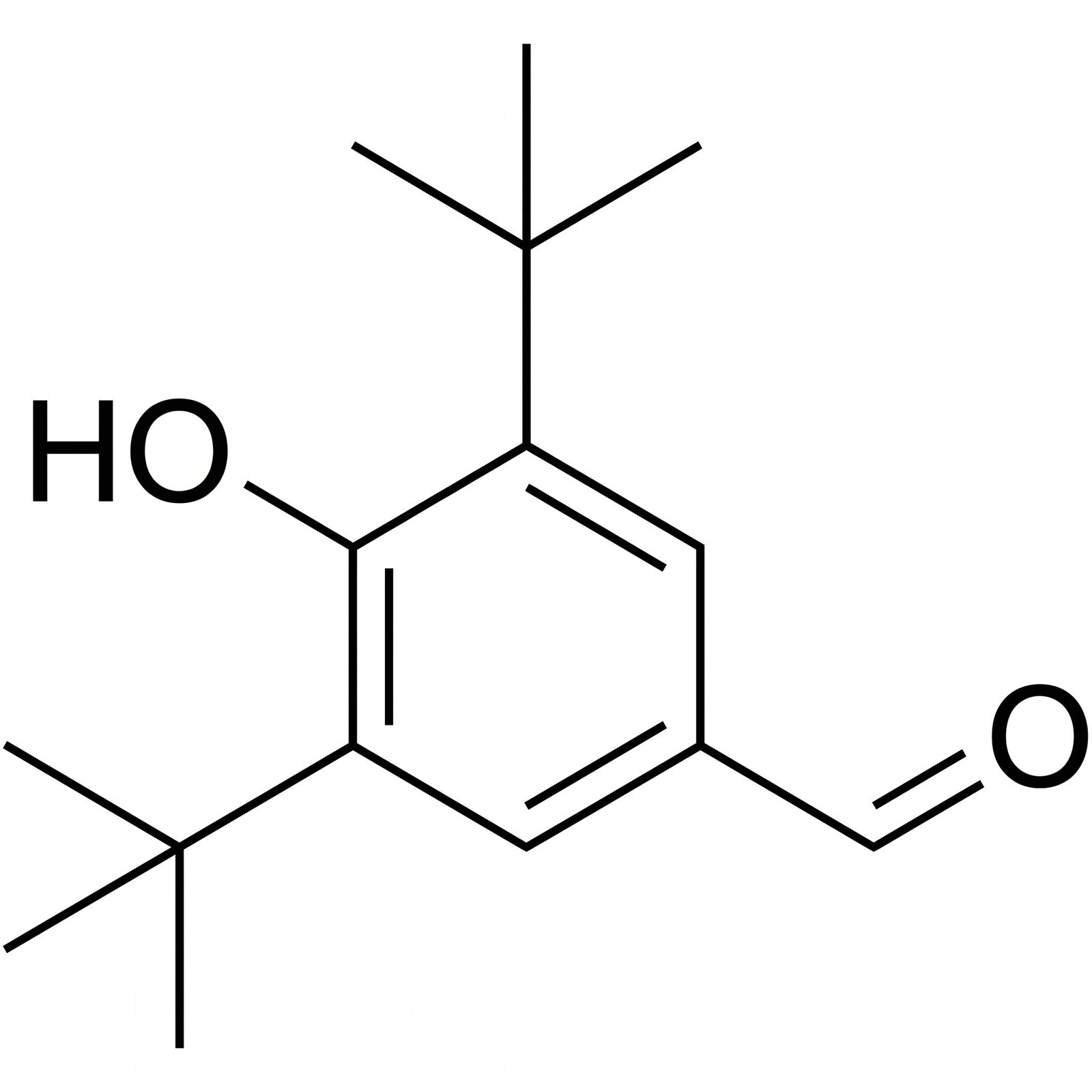	28.4	164	4.2	10.6	0.50
2，6-Di-*tert*-butyl-4-carboxyphenol	BHT-COOH	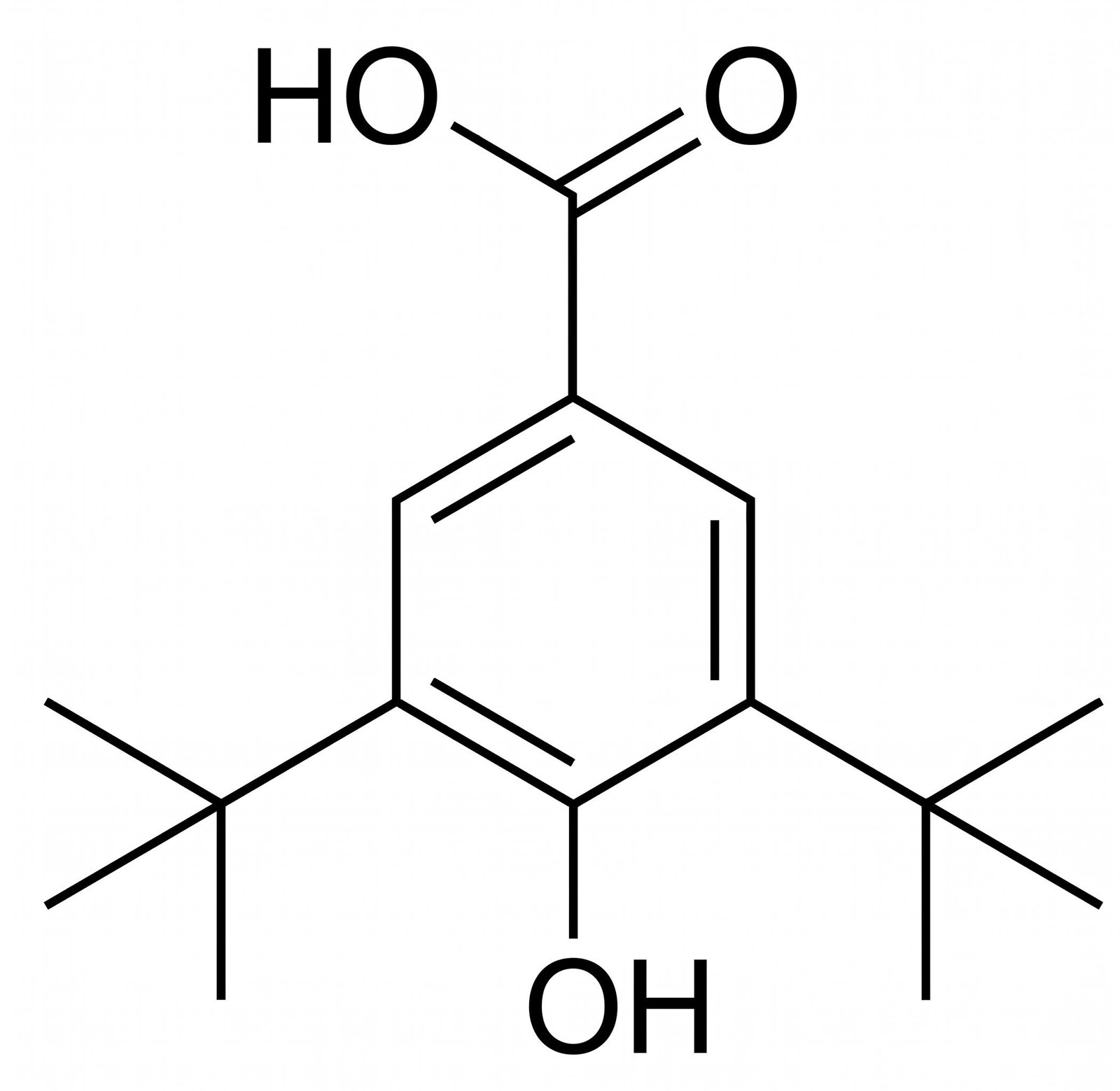	16.8	3.16	4.36	12.87	3.18
2，6-Di-*tert*-butyl-4-hydroxy-4-methylcyclohexa-2，5-dien-1-one	BHT-OH	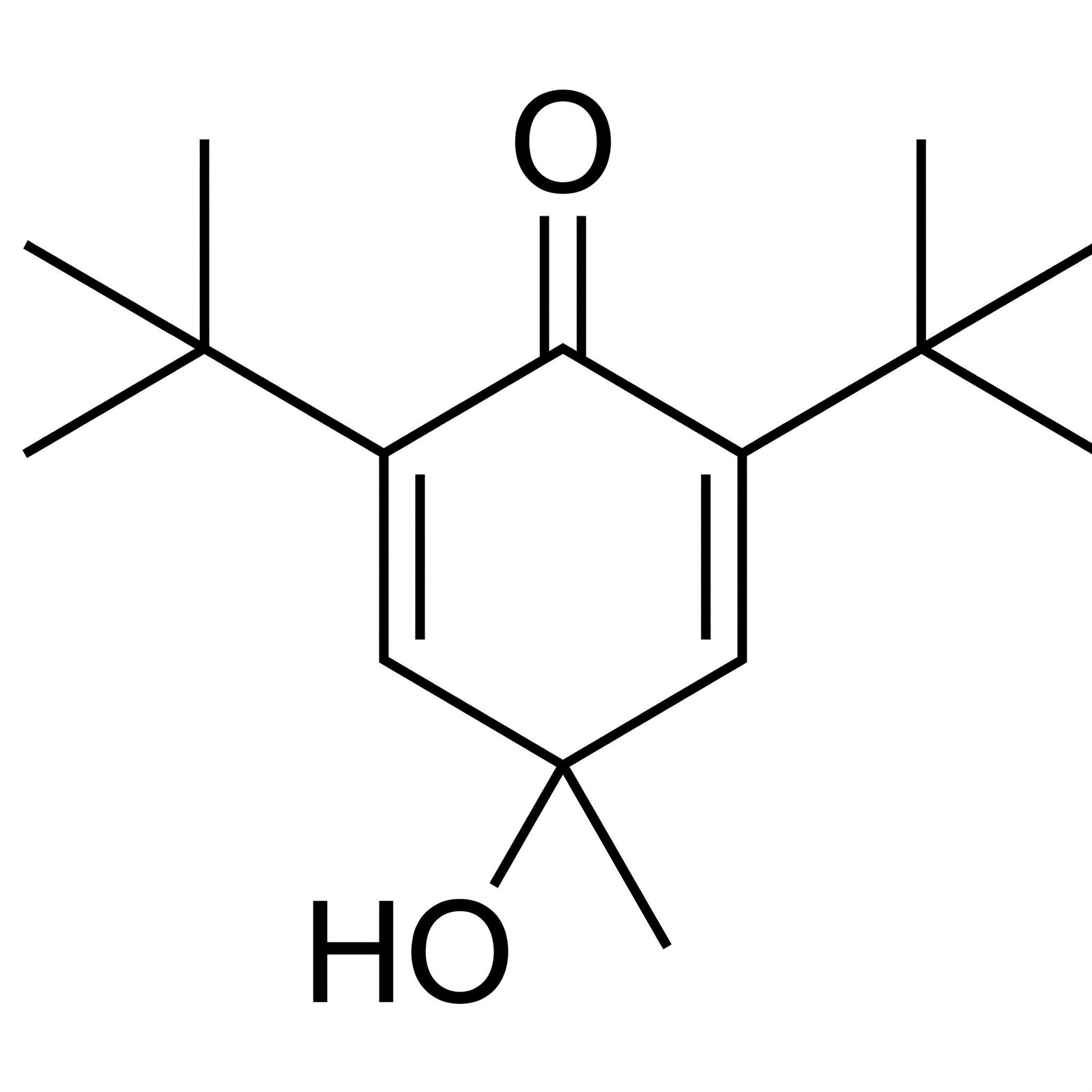	60.7	132	3.72	10.24	0.17
2，4-Di-*tert*-butyl-phenol	DBP	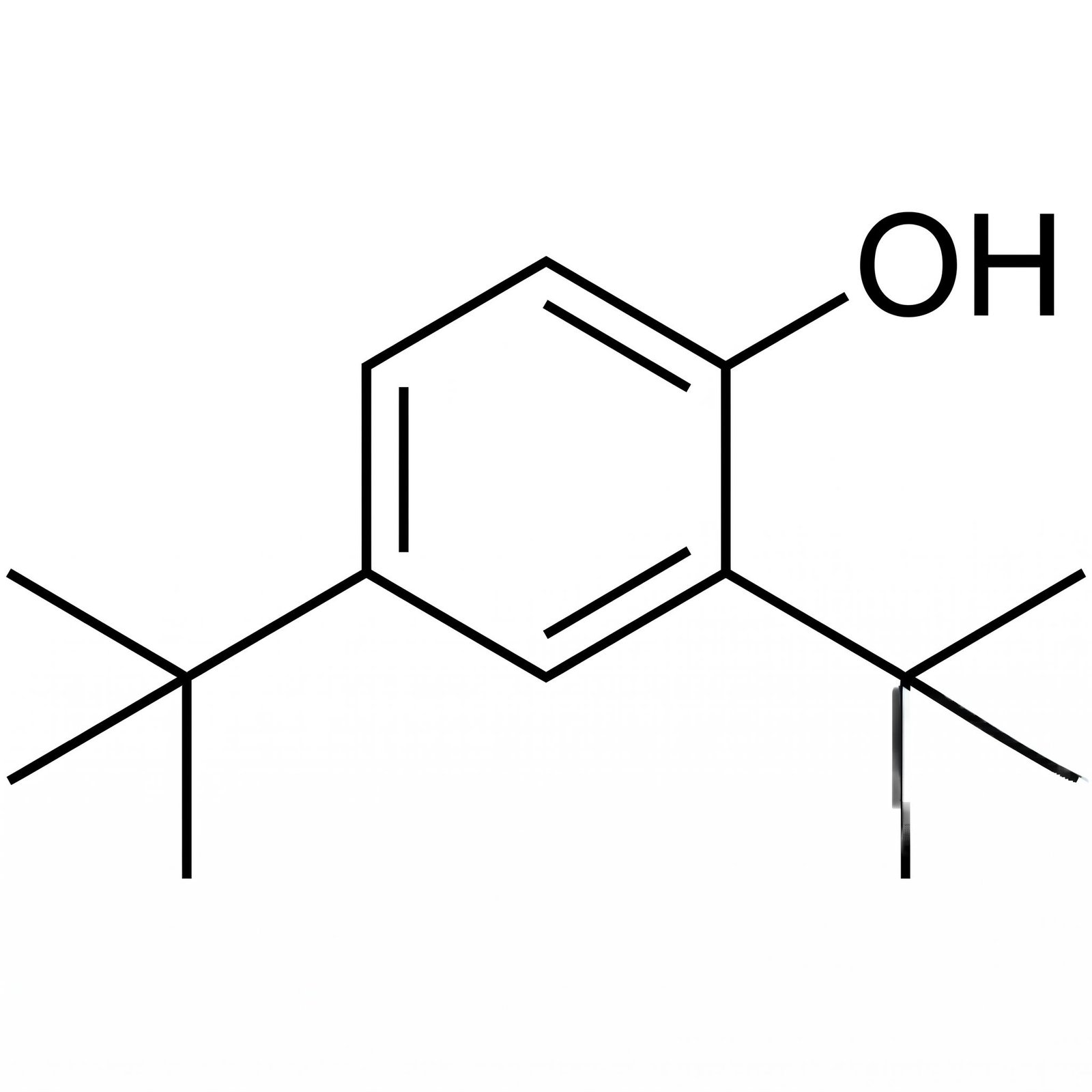	5.7	740	5.33	9.01	0.22
Dodecyl 3，4，5-trihydroxybenzoate	DG	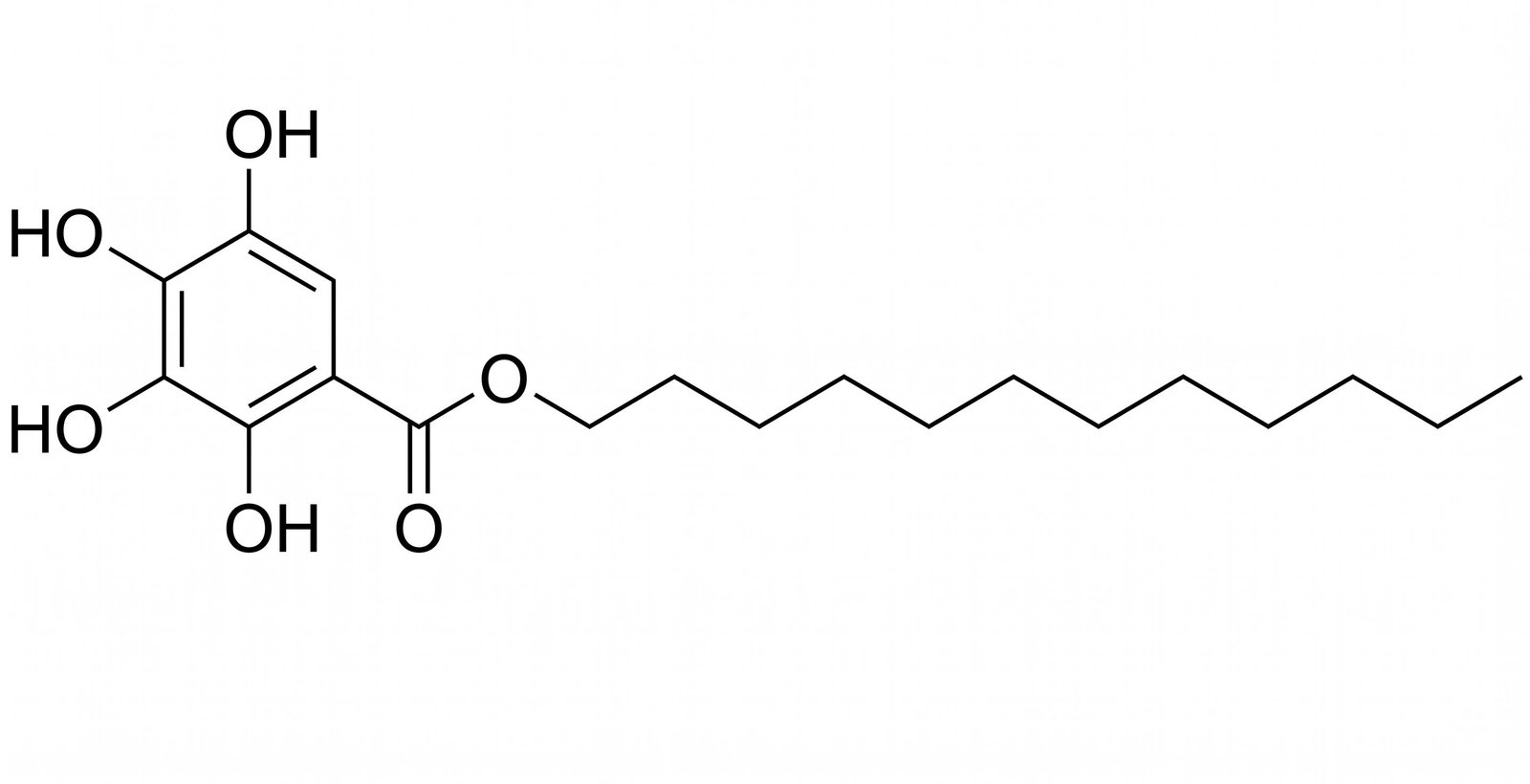	0.042	246	6.21	19.70	0.10
*tert*-Butylhydroquinone	TBHQ	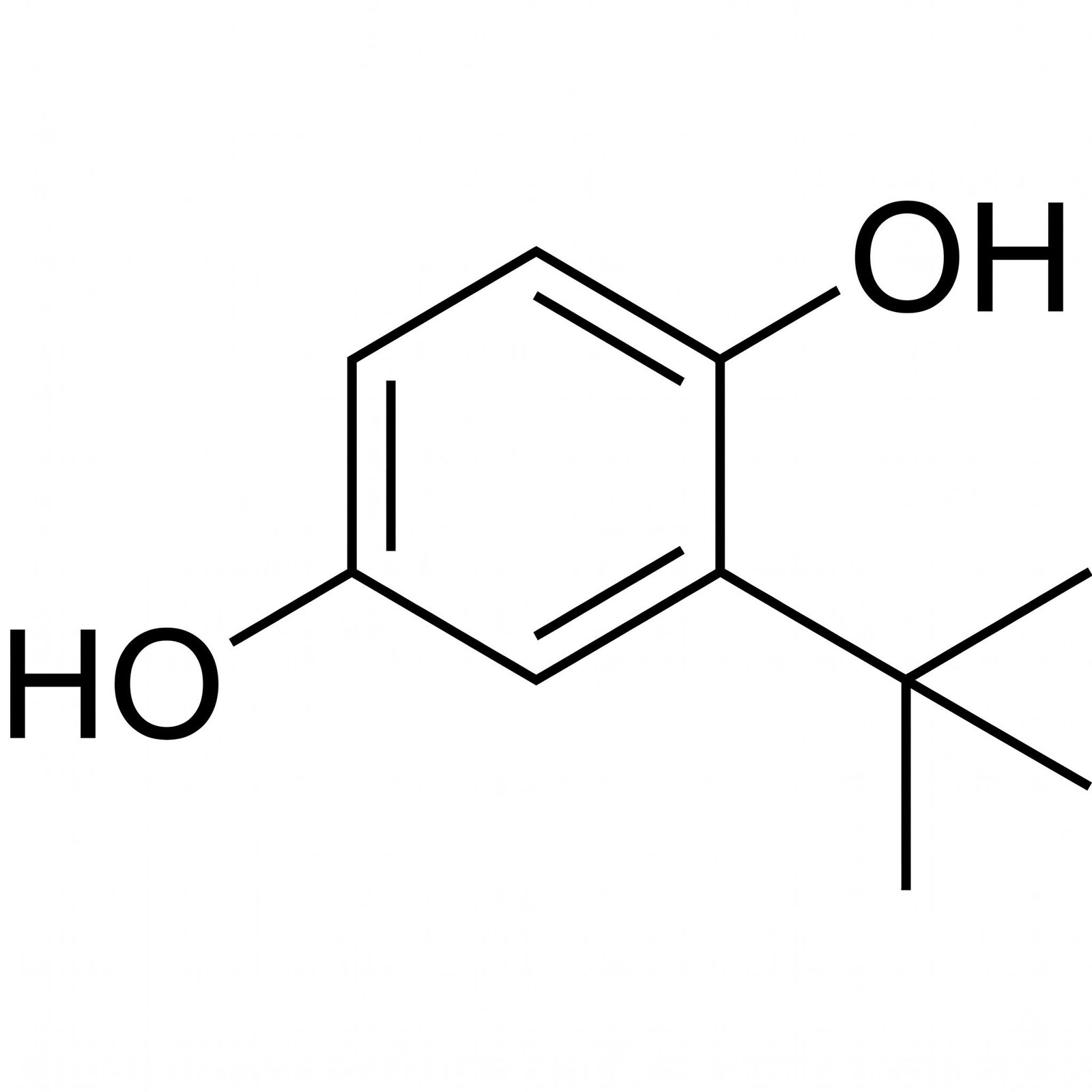	748	24.3	2.84	11.10	0.21
Pentaerythritol tetrakis（3-（3，5-di-*tert*-butyl-4-hydroxyphenyl）propionate）	AO1010	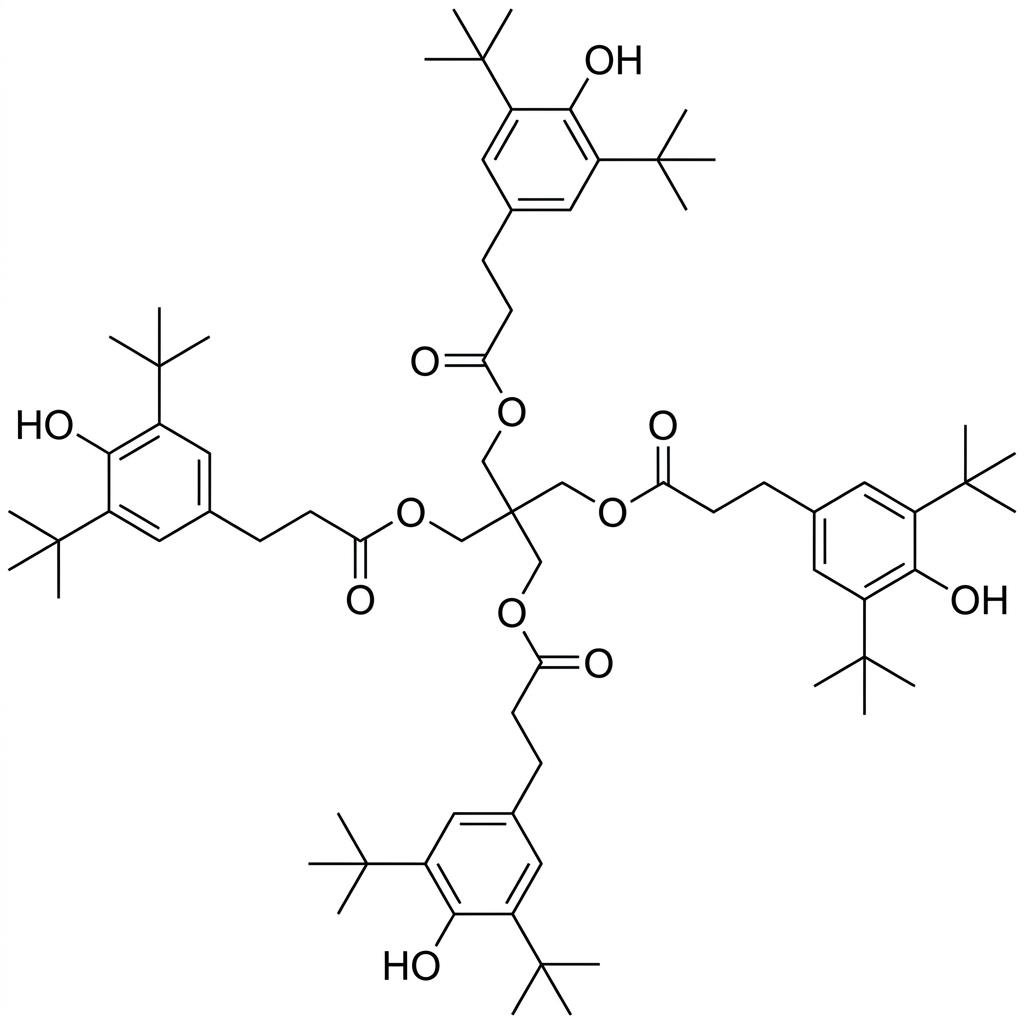	0.0052	2.2	19.60	27.40	0.10
*N，N′*-Bis［3-（3，5-di-*tert*-butyl-4-hydroxyphenyl）propionyl］hydrazine	AO1024	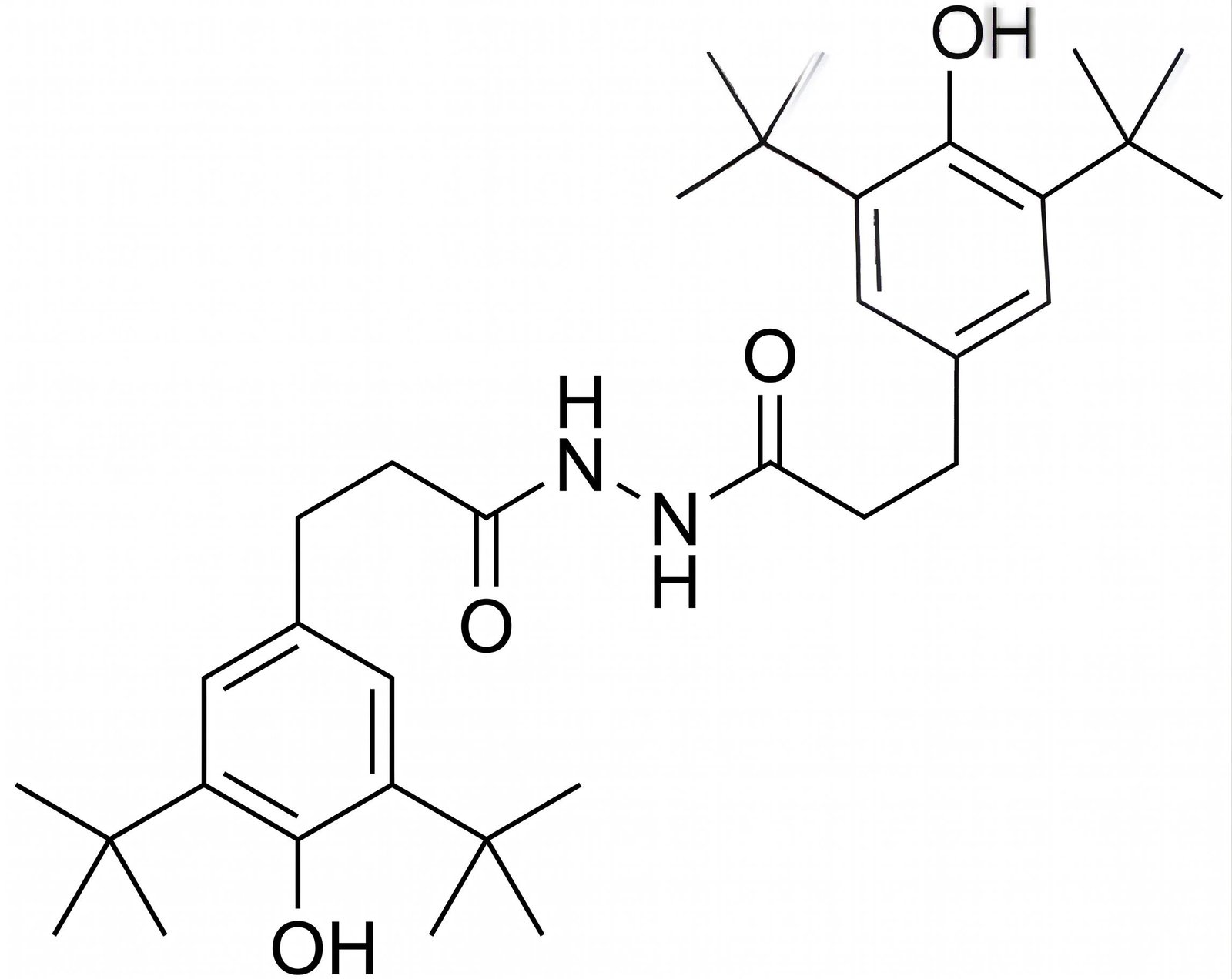	0.027	3264	7.79	24.1	0.20
Octadecyl-3-（3，5-di-*tert*-butyl-4-hydroxyphenyl）propionate	AO1076	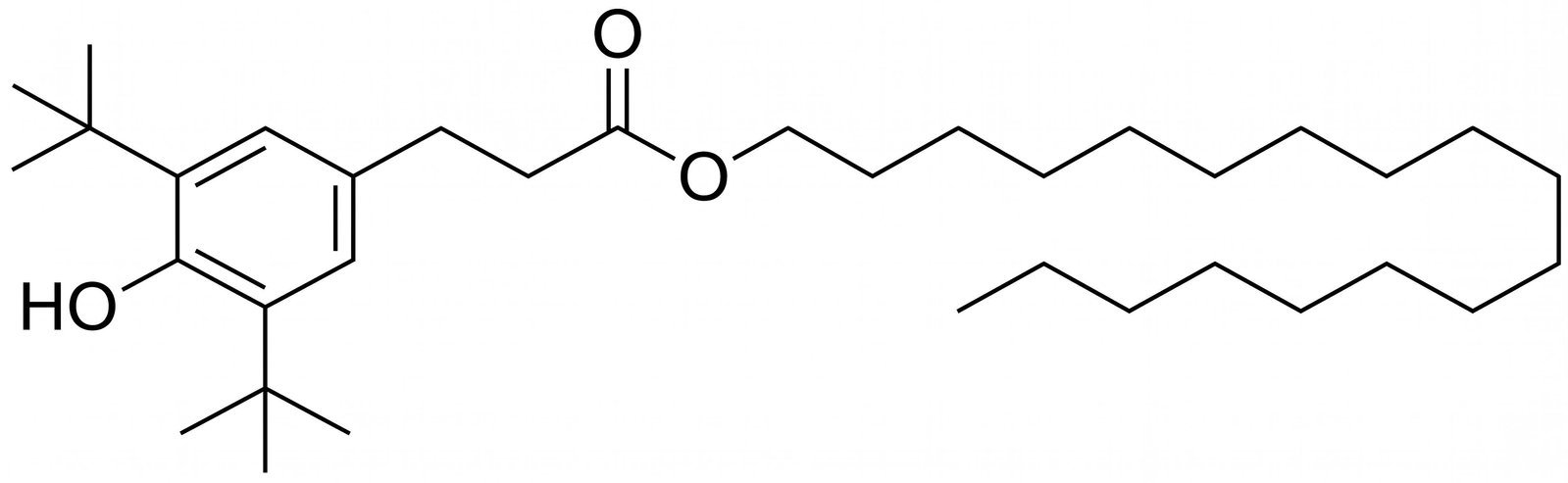	6.1×10^-9^	5.8	13.4	17.6	0.25
1，3，5-Tris（3*′*，5*′*-di-*tert*-butyl-4-hydroxybenzyl）isocyanuric acid	AO3114	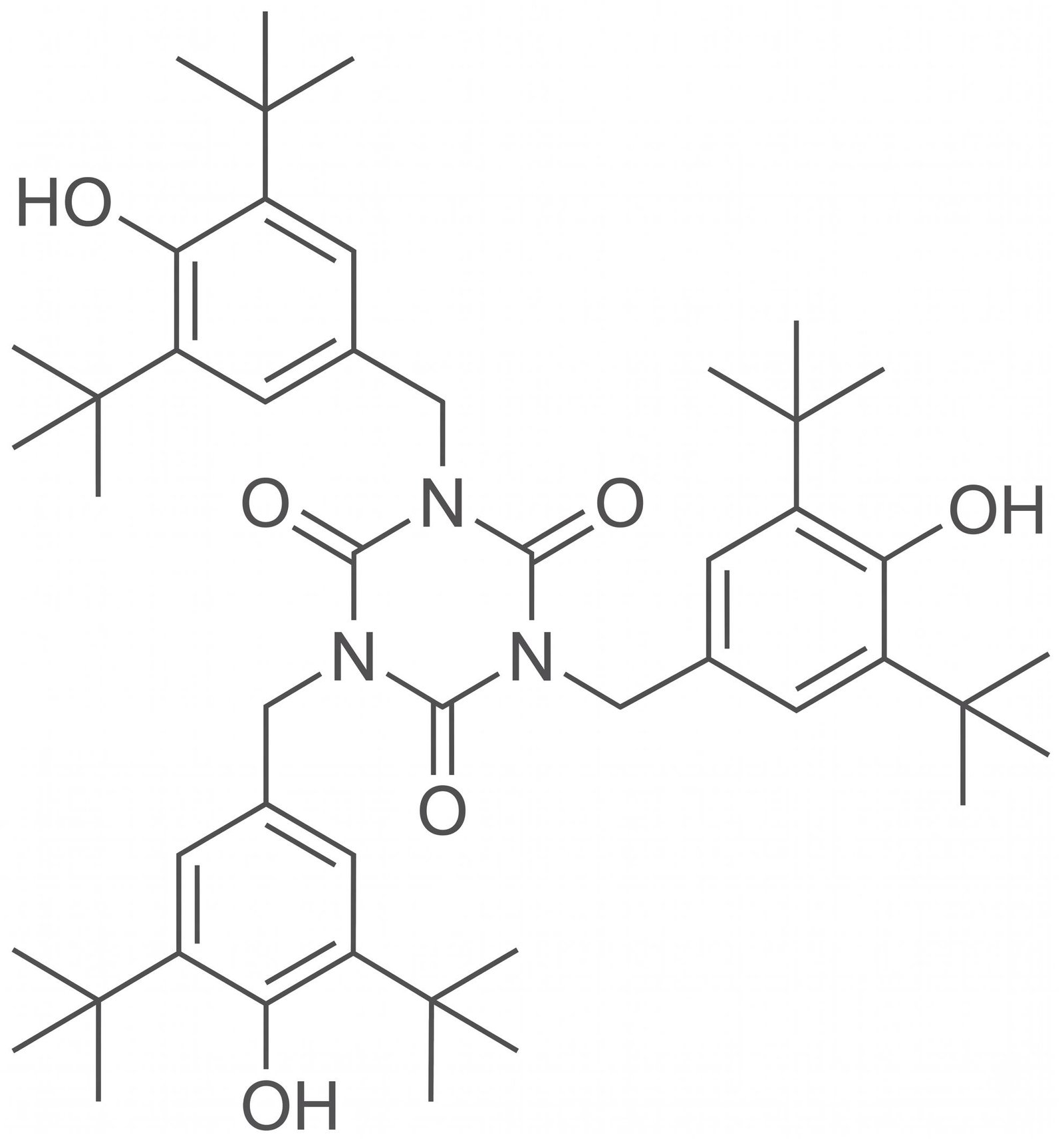	4.0×10^-12^	3.16	15.18	39.35	0.16

* All physicochemical properties are obtained from EPI Suite^TM^. BCF values are predicted using a regression-based method.

环境分析方法学与环境赋存行为研究，为其环境健康与生态风险评价提供科学依据。

近年来，非靶向筛查技术飞速发展，新型SPAs在环境中被不断发现。然而，现有综述主要聚焦SPAs的环境检出与人体暴露、毒性以及环境风险，缺少近5年的最新认识和发现，尤其是分析方法学方面的综述不足^［2，4］^。为此，本综述重点总结了近5年SPAs最新研究发现，系统总结了环境样品中SPAs的分析方法学，并分析对比了现有的SPAs环境样品前处理技术。

## 2 合成酚类抗氧化剂的环境释放与赋存特征

人类使用后的无意识排放是环境中SPAs的首要来源。这些化学添加剂与聚合物之间没有化学键连接，在产品生命周期的各阶段都可能释放到周围环境中（图1）。由于低挥发性，SPAs会首先吸附在气溶胶和地面灰尘中。在污水处理厂和其他水体环境中，SPAs更易蓄积在污泥和沉积物中。此外，它们还可通过垃圾填埋、农用地膜降解、有机肥/污泥施用、污水灌溉和大气沉降等多种途径进入土壤中。土壤是环境中SPAs最主要的储存库。各环境介质中检出种类和优势污染物呈现出从传统SPAs向新型高相对分子质量SPAs（如AO1010、AO1076）演变的趋势。

**图1:**
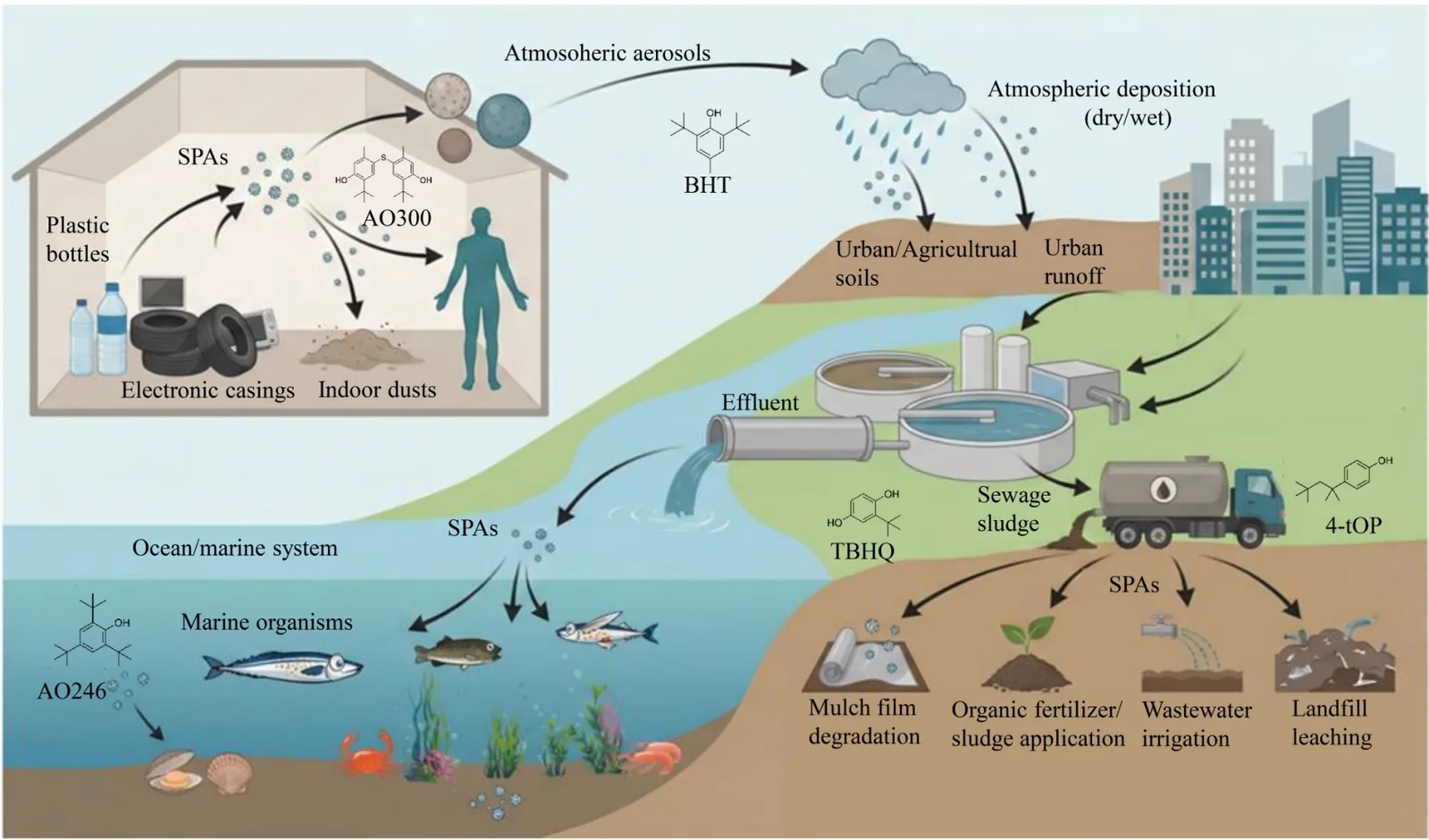
合成酚类抗氧化剂在环境中的释放和归趋行为

### 2.1 灰尘

大部分SPAs挥发性较低。它们在塑料或橡胶制品释放过程中会首先吸附在气溶胶表面，然后通过干湿沉降进入地面灰尘。因此，灰尘是环境中SPAs的首要暴露介质，尤其是室内灰尘。研究发现，室内灰尘中的SPAs浓度普遍高于室外灰尘^［5］^。BHT、BHT-Q及BHT-CHO（其化学结构见表1）是室内灰尘中检出最多的SPAs，且其在西方发达国家的浓度水平远高于中国^［5-9］^。例如，瑞典家庭灰尘中BHT浓度高达0.5~791 µg/g（平均浓度：70 µg/g）^［6］^，加拿大室内灰尘中BHT-Q最高浓度和几何平均浓度（GM）分别为4 770 ng/g和325 ng/g^［7］^。中国10省份城市室内灰尘中BHT和BHT-Q的GM值分别为11.5 ng/g和78.9 ng/g^［8］^。这种差异可能源于西方国家更长的SPAs使用历史和生活习惯差异（如空调使用率低）。此外，城市室内灰尘中SPAs浓度明显高于农村地区^［9］^。值得注意的是，AO1076、AO1010等一些新型SPAs在室内灰尘中被不断检出，且其浓度与家庭电子设备的数量呈正相关^［10］^。

各环境介质中合成酚类抗氧化剂的含量见表2。

**表2:** 各环境介质中合成酚类抗氧化剂的含量

Sample type	Sample source	DF range/%	Total concentrations	Dominant congeners	Ref.
House dust （*n=*83）	Toronto， Canada， 2018	11-100	∑_8_SPAs： 67.2-15540 ng/g （GM： 1490 ng/g）； ∑_4_TPs： 40.4-12700 ng/g （GM： 883 ng/g）	BHT： 658 ng/g （GM）； BHT-Q： 4770 ng/g （max）， 325 ng/g （GM）	［^7^］
Indoor dust （*n=*226）	10 provinces， China， 2018-2023	1.8-97	∑_6_SPAs： 15.4-3210 ng/g （GM： 169 ng/g， median： 149 ng/g）	BHT： 11.5 ng/g（GM）； BHT-Q： 78.9 ng/g （GM）	［^8^］
Indoor dust （*n=*75）	Jinan， China， 2014	urban： 16-100； rural： n.d. -100	urban： ∑_7_SPAs： 1560-20300 ng/g （GM： 3120 ng/g）；rural： ∑_7_SPAs： 668-4390 ng/g （GM： 1970 ng/g）	BHT （GM）： 2270 ng/g （urban）， 1700 ng/g （rural）； AO246 （GM）： 229 ng/g （urban）， 323 ng/g （rural）	［^9^］
Indoor dust （*n=*80）	Toronto， Canada， 2018	4-95	∑_10_SPAs： 239-213000 ng/g （GM： 1770 ng/g）	BHT： 749 ng/g（GM）	［^10^］
Sewage effluent （*n=*3）	Beijing， China， 2014	100	∑_9_SPAs： 1500 ng/L （mean）	BHT： 262 ng/L （mean）； BHT-CHO： 534 ng/L （mean）； BHT-Q： 108 ng/L （mean） BHT-quinol： 556 ng/L （mean）	［^11^］
Sewage sludge （*n=*56）	33 cities， China， 2010-2011	1.8-100	∑_14_SPAs： 183-41000 ng/g （mean： 4960 ng/g）	BHT： 4140 ng/g （GM）； AO246： 374 ng/g （GM）； BHT-Q： 562 ng/g （GM）	［^11^］
River water （*n=*9）	Germany， Oder River， 2000-2001	100	∑_2_SPAs： 88-520 ng/L （GM： 256 ng/L； mean： 281 ng/L）	BHT： 178 ng/L （mean）； BHT-CHO： 102 ng/L （mean）	［^12^］
Surface water （*n=*23）	PRD， China， 2024	4.5-100	∑_4_SPAs （median）： 95 ng/L （dry seasons）/42 ng/L （wet seasons）	BHT-COOH， BHT-OH	［^13^］
Surface water （*n=*32）	YRD， China， 2024	25-100	∑_18_SPAs： 2700 ng/L （GM）	AO3114： 1420 ng/L （median）	［^14^］
Sediments （*n=*32）	YRD， China， 2024	49-100	∑_18_SPAs： 1270 ng/g （GM）	AO1010： 609 ng/L （median）	［^14^］
Soils （*n=*32）	YRD， China， 2024	n.d.-100	∑_18_SPAs： 2440 ng/g （GM）	AO1010： 794 ng/L （median）； AO1076： 1600 ng/L （median）	［^14^］
Sewage sludge （*n=*45）	6 cities， China， 2017-2018	49-100	∑_10_SPAs： 94.6-6024 ng/g	AO246： 547 ng/g （GM）； AO1010： 212 ng/g （GM）； AO1076： 35.2 ng/g （GM）	［^15^］
Dumping site soils （*n=*12）	6 Asian countries， 2006 and 2015	100	∑_1_SPAs： 3.2-3300 ng/g	BHT： 3300 ng/g （max）	［^16^］
Human serum （*n=*50）	Temecula， CA， US， 2016-2017	4-100	∑_5_SPAs： 0.46-34.7 µg/L （GM： 7.77 µg/L） ∑_4_BHT-metabolites： n.d.-3.66 µg/L （GM： 0.77 µg/L）	BHT： 2.66 µg/L （GM）； 2，4-DTBP： 3.65 µg/L （GM）	［^17^］
Human urine （*n=*60）	Fort Lauderdale and Miami， US， 2018	30-100	∑_3_SPAs： 6.19-70.2 µg/L （GM： 18.6 µg/L）； ∑_4_BHT-metabolites： 0.54-8.61 µg/L （GM： 1.68 µg/L）	BHT-COOH： 0.68 µg/L （GM）； 2，4-DTBP： 18.3 µg/L （GM）	［^18^］
Human blood （*n=*30）	Stockholm， Sweden， 2020	17-100	∑_13_SPAs： 333-896 ng/g （median： 594 ng/g）	AO2246： 520 ng/g （median）； 4-tOP： 14 ng/g （median）	［^19^］
Breast milk （*n=*80）	Guangzhou， China， 2020	34-100	∑_8_SPAs： 1.96-64.9 µg/L （GM： 14.7 µg/L）； ∑_4_BHT-metabolites： 0.21-5.55 µg/L （GM： 1.83 µg/L）	BHT： 4.48 µg/L （GM）；2，4-DTBP： 9.94 µg/L （GM）	［^20^］

PRD： Pearl River Delta； YRD： Yangtze River Delta； GM： geometric mean； n.d.： not detected； DF： detection frequency in samples.

### 2.2 水体

除直接释放到地表灰尘外，SPAs还可通过污水处理厂出水间接排放到水体环境中。因此，污水处理厂是SPAs的重要环境排放途径之一。我国北京某污水处理厂出水中BHT的平均检出浓度为262 ng/L，而其代谢产物BHT-CHO和BHT-quinol的检出浓度更高，分别为534 ng/L和556 ng/L^［11］^。尤其值得关注的是，BHT-Q具有更长的半衰期（172 d，见表1）以及潜在的遗传毒性，其在出水中的浓度（108 ng/L）较进水显著增加了365%^［11］^。

SPAs在环境水体中同样被广泛检出。在德国Oder河等水域中，BHT和BHT-CHO的平均检出浓度分别为178 ng/L和102 ng/L，雨水中也有相当浓度的BHT（308 ng/L）和BHT-Q（155 ng/L）^［12］^。2024年在中国珠江三角洲地表水的研究显示，∑_4_SPAs在枯水期（中位值：95 ng/L）显著高于丰水期（42 ng/L），且主要污染物已由传统的BHT转变为其代谢物BHT-COOH和BHT-OH^［13］^。在长江三角洲水体中，一种新型SPA——AO3114被识别为主要污染物，GM值高达1 420 ng/L^［14］^。

### 2.3 沉积物、污泥和土壤

与水体相比，SPAs更易蓄积在污泥和沉积物中。一项涉及中国45座污水处理厂的2010-2011年污泥监测结果表明，我国污泥中∑_14_SPAs的最高浓度可达41.0 µg/g，其中BHT检出最多。其GM浓度（4.14 µg/g）显著高于欧美国家（1.2 µg/g）^［11］^。然而，在2017-2018年的全国污泥监测研究中，AO1010、AO1076及双（3，5-二叔丁基-4-羟基-苯丙酰）肼（AO1024）等10种新型SPAs被检出，其中AO246（GM值：547 ng/g）和AO1010（GM值：212 ng/g）检出最多^［15］^。类似地，在长江三角洲沉积物中，AO1010的检出率和浓度（中位值：609 ng/g）均占据主导地位^［14］^。这些研究表明新型高相对分子质量SPAs已成为污泥和沉积物中优势污染物。

研究还发现，BHT等SPAs在农田土壤和垃圾填埋场周边土壤中被普遍检出，最高浓度达3 300 ng/g^［16］^。美国EPI Suite软件预测结果亦表明，土壤是环境中合成酚类抗氧化剂最主要的储存库（47%~90%）。在长三角地区的土壤调查中，AO1076和AO1010均100%检出，GM值分别为1 600 ng/g和794 ng/g ^［14］^，表明其比传统SPAs具有更强的土壤蓄积性。

### 2.4 生物样品

人类可以通过膳食摄入、呼吸道吸入、表皮接触、母乳喂养等多种途径广泛暴露于SPAs。大量研究证据表明，SPAs在人体血液、尿液和母乳等生物样品中被频繁检出。在50例美国普通人群血清样本中，∑_5_SPAs的最高浓度可达34.7 µg/L，其中BHT和2，4-二叔丁基苯酚（2，4-DTBP）检出最多，GM值分别高达2.66 µg/L和3.65 µg/L^［17］^。在60例美国人体尿液样品中，BHT-COOH和2，4-DTBP为主要同系物，其GM值分别高达0.68 µg/L和18.3 µg/L^［18］^。最近，Engelhardt等^［19］^在瑞典人体血液中检出了高浓度2，2′-亚甲基双（4-甲基-6-叔丁基苯酚）（AO2246，中位值：520 ng/g）及多种新型SPAs。该研究还发现，女性尿样中BHT和BHA的浓度显著高于男性尿样，这可能与女性使用个人护理品频率更高有关^［19］^。在中国华南地区采集的80例母乳样品中，2，4-DTBP和BHT是主要的污染物，GM值分别为9.94 µg/L和4.48 µg/L^［20］^。鉴于婴幼儿较高的灰尘接触概率和独有的母乳暴露途径，婴幼儿是SPAs暴露的高危人群。

### 2.5 合成酚类抗氧化剂的环境生态风险

SPAs在进入环境后可对哺乳动物和鱼类产生不利影响^［21-30］^。研究发现，雄性大鼠在急性高剂量（0.5~1.0 g/kg）BHT暴露下可出现肾脏和肝脏损伤，短期重复暴露于相近剂量BHT则对雌雄大鼠均产生肝毒性^［21］^。较低剂量（25 mg/kg）的BHT亚慢性暴露（经口饲喂或腹腔注射）也能导致大鼠肝脏重量增加以及关键肝酶活性降低^［21］^。小鼠暴露于叔丁基对苯二酚（TBHQ）会诱导其巨噬细胞中谷胱甘肽耗竭和外源性代谢异常，导致炎症因子基因表达下调^［22］^。3-丁基羟基茴香醚（3-BHA）暴露可以导致小鼠体重增加，并通过诱导细胞脂质过量堆积干扰脂质代谢平衡；高脂饮食会显著加剧该效应^［23］^。此外，国际癌症研究机构基于充分的动物实验证据认定BHA具有潜在致癌性^［24］^。BHT经过生物转化或非生物转化后毒性明显增强^［25，26］^。具体而言，BHT-OH、BHT-SCH_3_、BHT-OOH和BHT-Q对小鼠的急性毒性（LD_50_：190~2 270 mg/kg）均强于母体BHT（LD_50_：约3 550 mg/kg）^［25］^。其中，BHT-COOH对小鼠皮肤具有肿瘤启动活性；BHT-Q可能具有遗传毒性^［26］^。体外研究证据表明，低至10^-6^ mol/L的BHT-Q可诱导细胞产生氧自由基，导致DNA裂解^［26］^。

SPAs在生态毒性方面的研究相对较少，但近年来的数据表明其生态风险不容忽视^［27-30］^。研究发现，从塑料容器析出的3，5-二叔丁基-4-羟基肉桂酸（一种AO1010的降解产物）和2，4-DTBP可降低斑马鱼胚胎孵化率、延缓幼鱼心跳，并具有神经毒性^［27，28］^。0.02~0.2 mg/L BHT会抑制双壳类动物的总血细胞计数、吞噬活性和溶酶体膜稳定性，导致其免疫功能下降^［29］^。最新的毒理学研究表明，在环境相关浓度下，BHT、BHT-COOH、BHT-Q均能显著增加斑马鱼幼虫巨噬细胞和中性粒细胞数量，降低其对副溶血性弧菌的抗菌抵抗力，并诱导产生氧化应激^［30］^。其中，BHT-COOH和BHT-Q的免疫毒性均显著强于母体化合物BHT^［30］^。在长三角水体中，*β*-（3，5-二叔丁基-4-羟基苯基）丙酸异辛醇酯（AO1135）和BHT-Q对藻类、溞类和鱼类的风险熵值均大于1，表明它们具有较高生态风险，并且它们的联合毒性风险随营养级升高而增加^［14］^。

## 3 合成酚类抗氧化剂的环境样品前处理与分析方法

各种方法学因素（如样品采集、前处理效率）可显著影响SPAs环境浓度定量结果。例如，在相同地区的室内灰尘中BHT的报道浓度差异可达数个数量级（表2）。这种差异有以下原因：（1）SPAs在环境介质中的浓度通常在痕量水平（ng/L或ng/g级）；（2）环境基质（如污泥、生物组织）非常复杂；（3）SPAs理化性质差异大（从极性代谢物到超疏水性新型SPAs），极易在分析过程中发生氧化降解或吸附损失；（4）不同分析方法中SPAs的检出限和定量能力差异大。因此，建立高效的前处理方法与高灵敏的仪器检测方法是研究SPAs环境赋存特征的关键之一。

### 3.1 环境样品前处理方法

不同前处理方法的提取效率、基质效应和操作复杂度差异明显。一般而言，液液萃取、固相萃取（SPE）和超声辅助萃取（UAE）简单高效。分散固相萃取（dSPE）方法开发复杂但灵活、可定制。加速溶剂萃取（ASE）提取效率较高，但带来的基质效应也较强，需要额外的净化步骤。SPAs前处理方法的选择还应考虑环境基质类型。液液萃取和SPE适用于水样和人体生物样品，UAE和ASE则适用于基质效应较强的非生物固体样品。

#### 3.1.1 水体样品

水样前处理方法主要是液液萃取和SPE。Tang等^［31］^采用甲基叔丁基醚液液萃取结合硅胶柱净化测定河水和污水处理厂进水中11种SPAs，加标回收率分别为65.8%~117%和59.5%~118%。由于新型SPAs具有极高的疏水性（log *K*_ow_>10），而BHT代谢物具有较强亲水性，SPE方法需要选择能同时保留不同极性化合物的萃取材料。HLB柱因其对宽极性范围化合物的优异保留能力而被广泛应用。Zhang等^［32］^通过优化HLB柱萃取条件，以甲醇和乙酸乙酯分步洗脱，成功建立了地表水中BHT、BHA以及5种BHT代谢物的痕量检测方法（定量限1.93~3.10 ng/L），回收率为76.0%~116%。此外，固相微萃取（SPME）技术也被用于检测挥发性较强的BHT^［33］^。Tombesi等^［33］^利用涂覆聚二甲基硅氧烷的SPME纤维结合GC-MS在顶空模式下检测瓶装水中的BHT，有效避免了溶剂背景干扰。

#### 3.1.2 非生物固体样品

土壤、沉积物及灰尘等固体样品基质效应强，前处理方法通常采用UAE。例如，Weng等^［8］^采用二氯甲烷-正己烷（1∶1，体积比）超声萃取灰尘中的SPAs，提取液经氮吹浓缩及溶剂置换后进行分析。该方法结合同位素稀释定量法显著提升SPAs及其羟基化/羧酸化代谢物的检测准确度，回收率为90.5%~105%，定量限低至0.21 ng/g。Zhang等^［34］^在UAE后增加了特异性衍生化步骤，使用BSTFA试剂将极性代谢物（如BHT-OH）转化为挥发性硅烷化产物。该方法显著提升了GC-MS的检测灵敏度，在沉积物样品中的检出限达0.02~0.34 ng/g。然而，常规UAE消耗大量溶剂且费时。Liu等^［11］^利用ASE技术的高温高压（90 ℃、1 500 psi）原理使污泥样品与提取溶剂充分接触，大大提高了提取效率、减少了溶剂消耗，回收率为65%~103%。

#### 3.1.3 生物样品

生物样品（如动植物组织、血液、母乳、尿液等）种类繁多，富含蛋白质和脂质。前处理和分析过程极易发生色谱柱堵塞和质谱基质效应。传统的除脂方法（如凝胶渗透色谱）步骤繁琐。对于动植物组织，Wang等^［35］^首先利用二氯甲烷-正己烷（3∶1，体积比）重复超声提取海洋软体动物体内的SPAs。提取液通过PTFE滤膜除脂，再利用HLB固相萃取柱进一步净化浓缩。方法回收率为61%~92%，定量限低至0.7 ng/g。Engelhardt等^［19］^在液液萃取后采用增强型脂质去除材料EMR-Lipid净化人血清样品；相比传统的Florisil柱^［20］^，EMR-Lipid能更特异性地吸附脂质而不损失疏水性SPAs。此外，人体生物样本中SPAs多以葡萄糖醛酸或硫酸结合态存在，直接提取方法只能测定游离态SPAs。Engelhardt等^［19］^采用*β*-葡萄糖醛酸酶/硫酸酯酶对人血清中结合态SPAs进行酶解预处理，将其转化为游离态SPAs，从而更准确地评估生物体的SPAs总内暴露水平。

### 3.2 仪器分析方法

#### 3.2.1 液相色谱-串联质谱（LC-MS/MS）

LC-MS/MS是目前新型大分子SPAs及极性代谢物的主流分析技术。然而，BHT等弱极性化合物在传统的电喷雾电离（ESI）源下电离效率极低。Du等^［36］^创新性地引入大气压化学电离（APCI）源，利用电晕放电产生的气态试剂离子使BHT发生电荷转移电离，在APCI负离子模式下，BHT的响应信号较ESI源提高约260倍，且能同时检测AO1010等大分子SPAs。此外，针对实验室环境中无处不在的SPAs背景干扰问题（可能来自塑料管路、流动相瓶盖等），Du等^［36］^在液相色谱泵后、进样器前串联了一根C18捕集柱。该捕集柱能将背景中的SPAs截留并延迟洗脱，使其与样品中的目标峰在保留时间上完全分离，从而实现痕量SPAs的“零背景”检测^［36］^。

#### 3.2.2 气相色谱-质谱（GC-MS）

GC-MS凭借其高分离度和标准谱库优势，仍是BHT、BHA等低相对分子质量挥发性SPAs的首选检测方法^［37］^。由于无流动相，GC-MS还可有效消除因流动相污染和管线释放造成的测量结果不准确问题。极性较强的代谢物（如BHT-COOH）在采用GC-MS分析时通常需要进行硅烷化衍生（如使用BSTFA或MSTFA）以增加其挥发性和热稳定性^［34］^。此外，GC-MS/MS由于具有更高的灵敏度，可以显著降低复杂基质的背景噪声，提高信噪比^［8］^。然而，GC-MS分析时间相比LC-MS/MS更长，效率较低。

#### 3.2.3 高分辨质谱与非靶向筛查

基于Q-TOF MS或Orbitrap MS的非靶向筛查（NTS）技术可以准确识别环境中新型SPAs及未知转化产物。有学者通过采集高精度的全扫描质谱数据，结合Kendrick质量亏损（KMD）分析和同位素模式匹配，成功识别出污泥中AO1010的水解产物Fenozan^［15］^以及生物体内BHT的谷胱甘肽结合物（GS-BHT）^［38］^。Liu等^［39］^建立了一种基于丹磺酰氯衍生结合质谱源内裂解的SPAs非靶向筛查技术。该NTS方法不仅大大提高了SPAs的检测灵敏度，还利用分子离子峰和特征碎片离子峰的固定质量差提高了筛查准确性。异构体分离也是SPAs分析的难点之一。例如，BHA存在2-丁基羟基茴香醚（2-BHA）和3-BHA两种异构体。Muchakayala等^［40］^采用Acquity BEH C18色谱柱结合精细优化的流动相（A为20 mmol/L磷酸盐缓冲液；B为20 mmol/L磷酸盐缓冲液-乙腈（3∶7，体积比），梯度洗脱）实现了2-BHA和3-BHA的基线分离，分离度为3。

综上，合成酚类抗氧化剂的前处理和仪器检测方法见表3。

**表3:** 合成酚类抗氧化剂各种前处理和仪器检测方法

Sample type	Target analytes	Preparation methods	Analysis methods	Recoveries/%	MDL	Ref.
Indoor dust	6 SPAs & 3 TPs	ultrasonication （DCM/hexane， 1∶1）	GC-MS/MS+LC-MS/MS （ESI）	90.5-105	0.21-22.5 ng/g （MQL）	［^8^］
Sewage Sludge	12 SPAs & 3 TPs	ASE （90 ℃， 1500 psi； DCM/hexane， 3∶1）	LC-MS/MS （ESI）	65-103	0.1-15 ng/g	［^11^］
Human serum	13 SPAs	protein precipitation+SPE （EMR-Lipid）	LC-MS/MS （ESI）+GC-MS	42-112	0.047-36.7 ng/g （MQL）	［^19^］
Human breast milk	10 SPAs & 4 TPs	ultrasonication （ACN/hexane， 3∶5）+SPE （Florisil）	LC-MS/MS （ESI）+GC-MS	58-95	0.006-1.2 ng/g （MQL）	［^20^］
River water and sewage water	11 SPAs	liquid-liquid extraction （MTBE）+silica gel column	LC-MS/MS （ESI）	river water： 65.8-117；sewage water： 59.5-118	river water： 0.3-0.9 ng/L；sewage water： 0.9-2.4 ng/L	［^31^］
Surface water	2 SPAs & 5 TPs	SPE （HLB）	GC-MS	76-116	1.93-3.10 ng/L （MQL）	［^32^］
Bottled drinking water	BHT	SPME （polydimethylsiloxane）	GC-MS （headspace injection）	84-119	4.2 μg/L	［^33^］
Sediment	BHT & TPs	ultrasonication （DCM/hexane， 3∶1）+derivatization	GC-MS	71-118	0.02-0.34 ng/g	［^34^］
Mollusks	9 SPAs & 4 TPs	ultrasonication （DCM/hexane， 3∶1）+SPE （HLB）	LC-MS/MS （ESI）+GC-MS/MS	61-92	0.7-1.9 ng/g （MQL）	［^35^］
Indoor dust	8 SPAs & 4 TPs	ultrasonication （DCM/hexane， 3∶1）	LC-MS/MS+ trap column （APCI+ESI）	67-98	0.002-0.09 μg/L （IDL）	［^36^］

TPs： transformation products； ACN： acetonitrile； ASE： accelerated solvent extraction； DCM： dichloromethane； MTBE： methyl *tert*-butyl ether； MDL： method detection limit； MQL： method quantification limit； IDL： instrumental detection limit.

## 4 总结与展望

SPAs是一类产量大、应用广的新污染物，已在水体、土壤、大气颗粒物及生物体等多种环境介质中被普遍检出，其污染特征正从传统的BHT向新型高相对分子质量SPAs（如AO1010、AO3114）演变。色谱-质谱联用技术和非靶向筛查技术可为其环境行为的解析提供有力工具。尽管当前研究已取得进展，但仍面临诸多挑战。首先，SPAs环境分析筛查方法亟待完善。SPAs理化性质差异大、化学性质活泼、极易被氧化，多残留同时分析存在一定的技术瓶颈。特别是现有的前处理技术往往忽略环境中以结合态形式存在的SPAs，极易低估环境浓度。其次，对转化产物与新型SPAs的风险认知不足。现有研究多关注BHT、BHA等传统SPAs，而对新型SPAs和毒性可能更强的转化产物（如BHT-Q、Fenozan）的环境归趋及长期生态风险缺乏系统评估。最后，环境行为与毒理学机制研究不够深入。目前关于SPAs及其转化产物在环境中的归趋尚不明确。在生态毒理方面，缺乏系统性的生态风险评估数据。相较于水生生物，陆生生态系统土著物种（如土壤无脊椎动物、陆生植物）的SPAs毒理学数据十分有限。

未来建议重点开展以下几方面的研究：开发抗氧化干扰的前处理技术与高分辨质谱联用方法，攻克复杂基质中痕量SPAs及其结合态的精准定量难题；加强对新型SPAs及其转化产物的非靶向筛查与识别，建立更完备的污染物清单；深入探究SPAs在“土壤-植物-微生物”系统中的迁移转化行为，重点关注其对蚯蚓等陆生土著生物的长期生态毒理效应，从而为SPAs生态风险的全面评估及管控标准制定提供科学依据。
